# Transplacental transfer efficiency of perfluoroalkyl substances (PFAS) after long-term exposure to highly contaminated drinking water: a study in the Ronneby Mother-Child Cohort

**DOI:** 10.1038/s41370-025-00758-2

**Published:** 2025-03-06

**Authors:** Erika Norén, Annelise J. Blomberg, Christian Lindh, Daniela Pineda, Kristina Jakobsson, Christel Nielsen

**Affiliations:** 1 Division of Occupational and Environmental Medicine, Department of Laboratory Medicine, Lund University, Lund, Sweden; 2 School of Public Health and Community Medicine, Institute of Medicine, Sahlgrenska Academy, University of Gothenburg, Gothenburg, Sweden; 3 Department of Occupational and Environmental Medicine, Sahlgrenska University Hospital, Gothenburg, Sweden; 4 Clinical Pharmacology, Pharmacy and Environmental Medicine, University of Southern Denmark, Odense, Denmark

**Keywords:** Drinking water contamination, Firefighting foam, PFAS hotspot, Early-life exposure

## Abstract

**Background:**

Perfluoroalkyl substances (PFAS) are stable chemicals used in various applications. PFAS exposure has been associated with lower birth weight and immunological effects in children, and limited evidence further suggests adverse neurodevelopmental effects. Previous studies show that PFAS cross the placental barrier during pregnancy leading to prenatal exposure of the fetus. Research on the transplacental transfer efficiency (TTE) of PFAS in highly exposed populations is lacking.

**Objective:**

This study aimed to estimate the TTE of eight PFAS and three perfluorooctane sulfonic acid (PFOS) isomers in a birth cohort with a wide range of PFAS exposures and to investigate if maternal exposure level impacted the estimated TTE.

**Methods:**

The participants, most of whom had been exposed to PFAS-contaminated municipal drinking water, were recruited between 2015 and 2020 after the end of exposure. We collected maternal serum samples during pregnancy and at delivery, as well as umbilical cord serum. Serum samples were analyzed using liquid chromatography-tandem mass spectrometry (LC-MS/MS). TTE was estimated as the ratio of the PFAS concentration in cord serum to maternal serum. We used generalized additive mixed models accounting for maternal characteristics to assess if maternal exposure level (i.e., high, intermediate, or background) modified the estimated TTE.

**Results:**

The study included 200 dyads with matched cord and maternal serum samples. The exposure profile was dominated by perfluorohexane sulfonic acid (PFHxS) and PFOS. We observed the highest overall transfer efficiency for PFHxS (median TTE: 0.68) and the lowest for the *n*-PFOS isomer (median TTE: 0.33). Higher TTEs were observed for PFHxS and PFOS (total and isomers) in background-exposed dyads.

**Impact statement:**

In a birth cohort with a wide range of exposures to primarily PFOS and PFHxS from contaminated drinking water, we found that the transplacental transfer efficiencies (TTE) of eight PFAS and three PFOS isomers were of considerable magnitude. The highest TTE were observed for PFOA and PFHxS, and for branched PFOS isomers compared with linear. Although we observed slightly lower TTE in mother-child dyads with high and intermediate exposures compared with dyads with background levels of exposure, the considerable TTE in highly exposed mothers implies high absolute prenatal exposure in children in contaminated areas.

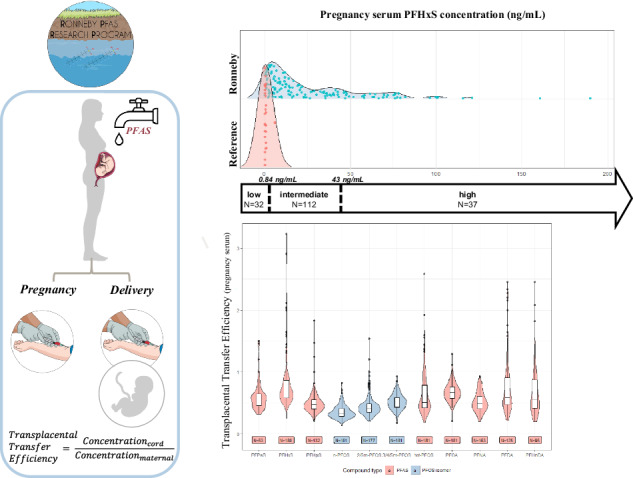

## Introduction

Perfluoroalkyl substances (PFAS) are synthesized organic chemicals with unique repelling characteristics which makes them ideal to use in a large variety of applications, including food packaging, non-stick materials, and aqueous film-forming firefighting foam (AFFF) [1]. These compounds have been used frequently since the 1950s, which has led to global environmental contamination.

In the last decade, it has been discovered that many areas close to firefighting training sites and airports are highly contaminated with PFAS due to the extensive use of AFFF in training activities [2–4]. Populations living near these sites are often exposed to PFAS from AFFF runoff contaminating water supplies [5–8]. Elevated serum levels of perfluorohexane sulfonic acid (PFHxS) and perfluorooctane sulfonic acid (PFOS) are strongly linked to AFFF exposure [9], as are elevated levels of less common compounds such as perfluoropentane sulfonic acid (PFPeS) and perfluoroheptane sulfonic acid (PFHpS) [5]. Ronneby, a municipality in southern Sweden, is one of the most impacted communities identified to date [10].

PFAS bioaccumulate and have endocrine-disrupting properties. They can cross the placental barrier [11–13], which leads to fetal exposure during sensitive periods of development. Evidence suggests that the transplacental transfer efficiency (TTE) varies by carbon chain length and functional group and that it differs between structural isomers of the same compound [11, 14–16]. Higher TTEs have been observed for branched PFOS isomers compared with linear PFOS [15, 17–20]. AFFF-related compounds, such as PFHpS, are less frequently measured in background-exposed populations, but results from a few studies indicate a TTE similar to other perfluorosulfonic acids (PFSA) [17–19].

General theories of transport of drugs or xenobiotics have been applied to explain the mechanisms of transplacental transport of PFAS [15, 18, 21–23], and both passive diffusion and active transport are likely involved in human placental tissue [21]. Free-state PFAS may be transported through passive diffusion, whereas protein-bound PFAS require an active transport process. Hence, variations in protein-binding affinity are thought to affect transferability [11, 24].

The current understanding of PFAS placental transfer is based on studies of the general population [11, 17, 25–27]. However, populations in communities exposed to high levels of PFAS have substantially higher serum concentrations than the general population, and it is not known whether maternal exposure levels affect TTE [11]. In this study, we estimated the TTE of eight PFAS compounds and three PFOS isomers in the Ronneby Mother-Child Cohort and assessed whether maternal exposure level modified the TTE.

## Materials and methods

### Setting

In December 2013, it was discovered that one of two municipal waterworks supplying drinking water to Ronneby municipality had extremely elevated concentrations of several PFAS [28]. One-third of the population had received contaminated water at their home address. The PFAS detected in the highest concentrations were PFOS and PFHxS, and elevated concentrations of perfluorohexanoic acid (PFHxA), perfluorobutane sulfonic acid (PFBS), perfluorooctanoic acid (PFOA), and PFHpS were also observed [10]. The contamination was caused by military training activities using AFFF at a nearby airfield since the 1980s. The contaminated waterwork was immediately shut down after the contamination was discovered. Biomonitoring of the population after the end of drinking water exposure showed a hundredfold higher serum concentrations of PFHxS and PFOS, and highly elevated concentrations of PFOA compared with a reference population sampled at the same time from the neighboring municipality Karlshamn with confirmed background levels of exposure [10].

### Ronneby Mother-Child Cohort

The Ronneby Mother-Child Cohort is a prospective birth cohort initiated as part of a comprehensive research program [29]. Between 2015 and 2020, all pregnant women in Ronneby were invited by their midwife to participate. In 2018, 35 pregnant women from Karlshamn were also enrolled to ensure enough participants with background levels of exposure. In total, 263 pregnant women between 18 and 43 years of age were recruited at their antenatal clinic.

### Sample collection

A venous maternal blood sample (2 × 5 mL) was collected during pregnancy (median gestational week 29, IQR 25 – 33) at a routine visit to the antenatal clinic. Umbilical cord blood (5–10 mL) and maternal blood (2 × 5 mL) were collected at the maternity ward at Blekinge Hospital at delivery. All blood samples were centrifuged at 3000 rpm for 10 min within 12 h from the collection to separate serum, which was transferred to 3 cryotubes. Serum samples were frozen to –70 °C before cold chain transport to our laboratory at Occupational and Environmental Medicine at Lund University. All samples were thereafter stored at –80 °C until analysis.

### Chemical analysis

The serum samples were analyzed for PFAS using liquid chromatography-tandem mass spectrometry (LC-MS/MS; QTRAP 5500 and 6500+; AB Sciex, Framingham, MA, USA) at the Division of Occupational and Environmental Medicine at Lund University according to Norén et al. [30]. Briefly, 25 µl serum samples were diluted with 25 µl water, added with isotopically labeled internal standards for all but PFPeS and PFHpS (Wellington Laboratories Guelph, Ontario, Canada), and solvents, and shaken vigorously for 30 min to precipitate proteins and centrifuged. The matched serum samples from each mother-child dyad were analyzed in 96-well plates in the same batch.

The total, non-isomer specific compounds PFBS, PFPeS, PFHxS, PFHpS, PFOS, perfluorodecane sulfonic acid (PFDS), perfluoropentanoic acid (PFPeA), PFHxA, PFHpA, PFOA, PFNA, PFDA, perfluorododecanoic acid (PFDoDA), PFUnDA, and perfluorotridecanoic acid (PFTrDA) were included in the analysis. At least two MS/MS transitions were included for each PFAS in the analytical method, except for PFHxA and PFHxS. For higher specificity in the analysis of PFHxS, the ratios between three transitions (399-80, 399-99, and 399-119) were evaluated. In an additional analytical method described by Xu et al. [5], PFOS was separated into linear PFOS (*n-*PFOS) and three separate isomer peaks. For the branched PFOS isomers, *2m-PFOS and 6m-*PFOS could not be separated and the sum of perfluoro-2/6-methylheptanesulfonate (*2/6m-*PFOS) was evaluated using a calibration curve of *6m-*PFOS. Likewise, the branched isomers *3m-*PFOS, *4m-*PFOS, and *5m-*PFOS could not be separated, and therefore the sum of perfluoro-3/4/5-methylheptanesulfonate (*3/4/5m-*PFOS) was evaluated using a calibration curve for *5m-*PFOS. The isomer perfluoro-1-methylheptanesulfonate (*1m-*PFOS) was omitted from further analysis because of suspected interferences.

The limit of quantification (LOQ) was defined as ten times the standard deviation of the concentrations in chemical blank samples and was 0.1 ng/mL for all PFAS. We estimated between-run precision using four quality control (QC) samples and between-batch precision by comparing duplicate samples for 25% of the samples above LOQ (Table S1). Details of the method are presented in the Supplementary Information. The laboratory participated successfully in the HBM4EU QA/QC program for PFAS analysis and participates bi-annually in the German External Quality Assessment Scheme (G-EQUAS) coordinated by the University of Erlangen-Nuremberg, Germany, for PFOA, PFNA, PFDA, PFBS, PFHpS, PFHxS, and PFOS analysis. A certificate is included in the Supplementary Information.

### Statistical analysis

We used Spearman’s rank correlation to assess bivariate correlations between each PFAS in maternal and cord serum. We calculated the TTE of each PFAS compound and PFOS isomer as the ratio of cord serum concentration to maternal serum concentrations for compounds above the LOQ in at least 30% of the maternal and cord samples:

$${TTE}=\frac{{{Conc}}_{{cord}}}{{{Conc}}_{{maternal}}}\times 100$$

TTE_mp:c_ was calculated using maternal concentrations in pregnancy, while TTE_md:c_ was calculated using maternal concentrations at delivery. For each compound or isomer, we excluded dyads for which the concentration in maternal serum or cord serum was below LOQ. We restricted the analysis to full-term pregnancies (≥37 gestational weeks) because previous research has demonstrated that TTE in preterm births is lower than that of full-term births [31].

We categorized maternal exposure into three levels based on the measured pregnancy serum concentrations of PFHxS. In our previous research, we found that PFHxS was the strongest marker of historical exposure to contaminated drinking water in Ronneby by comparing measured serum concentrations of PFAS with individuals’ residential history from the Swedish Total Population Register and municipal water-distribution data on the household level [10]. Women were categorized as background exposed if they had a serum concentration equal to or lower than the 90th percentile concentration of women living in Karlshamn (0.84 ng/mL; *n* = 26). The 90th percentile was chosen to avoid distortion of the true exposure profile in the reference municipality by classifying women who lived in Ronneby but received antenatal care in Karlshamn as background exposed. Women were categorized as highly exposed if they were at or above the 75th percentile concentration of PFHxS measured in all remaining women (43 ng/mL), an arbitrary cutoff chosen to create two sufficiently large categories with a clear exposure contrast while considering the distribution of data. Women who did not qualify for either of these categories were considered intermediate exposed. The distribution of maternal PFHxS exposure in the study participants in Karlshamn and Ronneby, including the exposure cut-offs, is shown in Figure S1.

To investigate whether TTE_mp:c_ depended on maternal exposure level, we stratified TTE by maternal exposure category for the AFFF-associated PFAS that were detectable in over 75% of cord blood samples (i.e., PFHxS, total and isomer-specific PFOS, and PFOA). Next, we evaluated whether TTE_mp:c_ was associated with maternal exposure using generalized additive mixed models with a random intercept for mothers to control for within-mother correlation of mother-child dyads. For each PFAS, we estimated the association between TTE_mp:c_ and exposure category both in unadjusted models and in models further adjusted for maternal characteristics that may potentially impact transplacental transfer (i.e., maternal age, pre-pregnancy BMI, parity, educational attainment, and smoking status in early pregnancy). TTE_mp:c_ values were log-transformed to improve the normality of residuals, and continuous variables were modeled as non-linear variables using penalized thin-plate splines. Models were limited to mothers with complete covariate information.

The statistical analyses were conducted in R version 4.4.0. Generalized additive mixed models were run using the package “mgcv” version 0.2-6 [32].

## Results

**Fig. 1:**
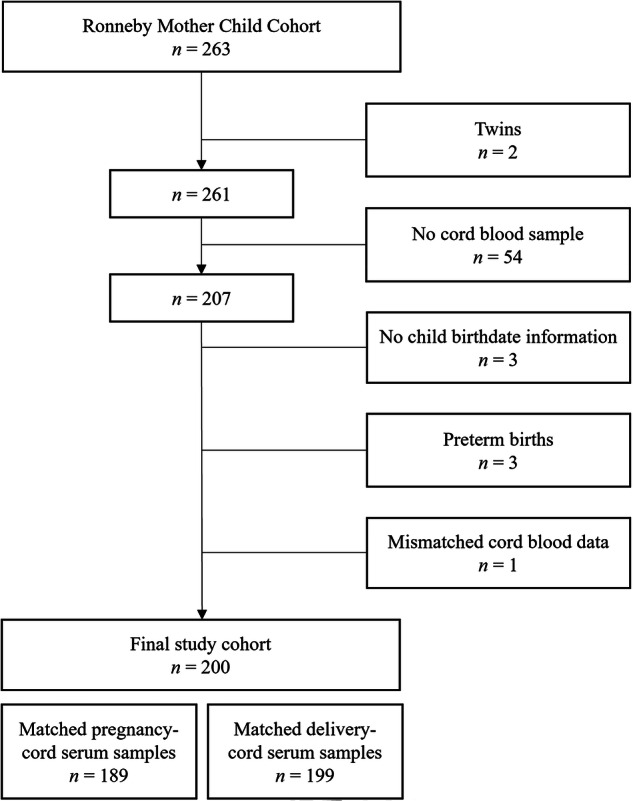
Flow chart describing the study sample.

In the cohort, 208 dyads provided at least one maternal serum sample and a cord serum sample. After excluding twin pregnancies, preterm births, and incomplete observations, the final study sample consisted of 200 dyads (Fig. 1).

Of the 189 women with a pregnancy serum sample, 38 were in the high exposure category, 113 were in the intermediate exposure category, and 38 were categorized as having background exposure.

### Descriptive statistics

The sociodemographic characteristics of the high and intermediate exposure categories were similar to the category with background exposure (Table 1), except that the highly exposed category had a higher proportion of primiparous women. As a result of the study recruitment process, the highly exposed category also had a larger proportion of deliveries in the early recruitment period. In the high- and intermediate-exposed category, women were more often smokers compared with the background-exposed category. No highly exposed participants received antenatal care in Karlshamn.

**Table 1:** Characteristics of the study sample, overall and stratified by maternal exposure category.

			Exposure category		
		Overall (*n* = 200)	High (*n* = 38)	Intermediate (*n* = 113)	Background (*n* = 38)
Antenatal care, location	Ronneby	171 (86)	38 (100)	110 (97)	12 (32)
	Karlshamn	29 (15)	0	3 (1)	26 (68)
Calendar year of delivery	2015	13 (7)	5 (13)	4 (4)	1 (3)
	2016	46 (23)	12 (32)	31 (27)	0
	2017	45 (23)	10 (26)	29 (26)	3 (8)
	2018	44 (22)	5 (13)	23 (20)	15 (40)
	2019	50 (25)	6 (16)	24 (21)	19 (50)
	2020	2 (1)	0	2 (2)	0
Maternal age, delivery (years)		30.6 (5)	30.3 (5)	30.7 (5)	31.0 (5)
Parity	Primiparous	76 (38)	21 (55)	40 (35)	12 (32)
	Multiparous	124 (62)	17 (45)	73 (65)	26 (68)
Maternal smoking status, early pregnancy	Non-smoker	180 (90)	34 (90)	103 (91)	35 (92)
	Smoker	10 (5)	2 (5)	8 (7)	0
	Missing	10 (5)	2 (5)	2 (2)	3 (8)
Pre-pregnancy BMI		25.5 (5)	25.5 (4)	26.0 (6)	24.5 (5)
Maternal educational attainment	Primary and lower secondary	4 (2)	0	3 (3)	0
	Upper secondary	79 (40)	15 (40)	45 (40)	16 (42)
	Post-secondary	107 (54)	21 (55)	63 (56)	19 (50)
	Missing	10 (5)	2 (5)	2 (2)	3 (8)
Gestational week, maternal pregnancy sample		29.4 (5)	28.6 (4)	28.6 (5)	32.2 (5)
	Missing	48 (24)	5 (13)	25 (22)	8 (21)

Maternal age at delivery, pre-pregnancy BMI, and gestational week of pregnancy sampling are presented as mean (standard deviation). All other parameters are presented as *n* (%).

### PFAS concentrations

The concentrations of the PFAS with a quantification rate of above 30% LOQ are presented in Table 2. PFBS, PFDS, PFPeA, PFHxA, PFDoDA, and PFTrDA were analyzed but not quantified as the levels were not discernible and they were therefore not further assessed. PFHpA was quantified in less than 8% of the serum samples.

**Table 2:** Overall concentrations (ng/mL) of PFAS in maternal serum in pregnancy and at delivery, and in cord serum in the study sample.

PFAS compound/PFOS isomer		Maternal pregnancy (*n* = 189)	Maternal delivery (*n* = 199)	Cord (*n* = 200)
PFPeS	%>LOQ^1^	36	33	36
	5th–95th P	<LOQ-1.07	<LOQ-0.86	<LOQ-0.58
	Median (Q1, Q3)	<LOQ (<LOQ, 0.20)	<LOQ (<LOQ, 0.15)	<LOQ (<LOQ, 0.12)
PFHxS	%>LOQ	100	100	100
	5th–95th P	0.30–78.7	0.30–69.1	0.42–53.8
	Median (Q1, Q3)	11.6 (1.75, 38.9)	10.6 (1.65, 32.1)	7.24 (1.35, 23.4)
PFHpS	%>LOQ	79	79	74
	5th–95th P	<LOQ-4.91	<LOQ-4.20	<LOQ-2.47
	Median (Q1, Q3)	0.73 (0.16, 2.32)	0.61 (0.12, 1.98)	0.36 (<LOQ, 1.05)
*tot-*PFOS	%>LOQ	100	100	100
	5th–95th P	1.87–112.0	1.89–96.3	1.84–51.2
	Median (Q1, Q3)	18.2 (4.89, 47.0)	15.8 (4.56, 41.9)	8.54 (3.79, 19.6)
*n-*PFOS	%>LOQ	100	100	100
	5th–95th P	1.41–65.8	1.27–55.4	0.62–21.4
	Median (Q1, Q3)	11.5 (3.30, 29.8)	9.94 (3.16, 23.7)	3.83 (1.23, 8.81)
*1m-*PFOS	%>LOQ	93	88	88
	5th–95th P	<LOQ-10.8	<LOQ-9.14	<LOQ-7.82
	Median (Q1, Q3)	1.35 (0.26, 4.37)	1.29 (0.24, 4.06)	1.10 (0.22, 3.24)
*2/6m-*PFOS	%>LOQ	100	100	98
	5th–95th P	0.29–14.5	0.25–12.3	0.13–6.02
	Median (Q1, Q3)	2.14 (0.65, 6.53)	1.97 (0.60, 5.06)	0.93 (0.31, 2.37)
*3/4/5m-*PFOS	%>LOQ	100	100	100
	5th–95th P	0.35–25.4	0.32–20.2	0.17–13.0
	Median (Q1, Q3)	4.34 (0.89, 11.9)	3.56 (0.80, 11.0)	2.02 (0.48, 5.69)
PFOA	%>LOQ	100	100	100
	5th–95th P	0.52–7.75	0.45–6.17	0.31–5.31
	Median (Q1, Q3)	1.82 (1.06, 3.58)	1.52 (0.96, 2.92)	1.25 (0.72, 2.34)
PFNA	%>LOQ	99	99	90
	5th–95th P	0.19–0.80	0.17–0.78	<LOQ-0.45
	Median (Q1, Q3)	0.42 (0.31, 0.57)	0.36 (0.26, 0.48)	0.19 (0.14, 0.27)
PFDA	%>LOQ	96	94	73
	5th–95th P	0.11–0.48	<LOQ-0.46	<LOQ-0.32
	Median (Q1, Q3)	0.25 (0.20, 0.32)	0.22 (0.15, 0.30)	0.14 (<LOQ, 0.19)
PFUnDA	%>LOQ	90	88	55
	5th–95th P	<LOQ-0.50	<LOQ-0.43	<LOQ-0.25
	Median (Q1, Q3)	0.21 (0.14, 0.30)	0.20 (0.13, 0.28)	0.10 (<LOQ, 0.15)

^1^LOQ = 0.1 ng/mL for all PFAS.

The results are restricted to PFAS detected at >30% above LOQ and higher.

All PFAS had a quantification rate above 75% in maternal pregnancy serum besides PFPeS, which had a quantification rate of 35%. PFAS levels were generally lower in cord serum compared with maternal serum. Total PFOS was detected in the highest concentration in both maternal and cord serum, followed by PFHxS. The linear PFOS isomer was found in higher concentrations than the branched isomers (Table 2).

In both maternal and cord serum, PFHxS concentrations were significantly correlated with the concentrations of other AFFF-related compounds including PFHpS and total PFOS (Fig. S2). Some PFAS compounds were strongly correlated between pregnancy and cord serum measurements, including PFPeS, PFHxS, PFHpS, total PFOS, and PFOA.

When we categorized mothers based on their pregnancy PFHxS concentration, we observed substantial exposure contrasts in PFOS (total and separate isomers), PFOA, and PFHpS concentrations in pregnancy serum (Table 3). PFPeS was quantifiable in most of the highly exposed pregnancy samples, but concentrations in the background and intermediate categories were mostly below the LOQ. Concentrations of PFNA, PFDA, and PFUnDA were low across all exposure categories. Concentrations of PFAS in maternal serum at delivery and in cord serum followed similar patterns when stratified by exposure category (Table S2).

**Table 3:** Maternal serum concentrations in pregnancy (ng/mL) in the high, intermediate, and background exposure categories.

		Exposure category		
PFAS compound/ PFOS isomer		High (*n* = 38)	Intermediate (*n* = 113)	Background (*n* = 38)
PFPeS	%>LOQ^1^	74	35	0
	Median (Q1, Q3)	0.29 (<LOQ, 0.78)	<LOQ (<LOQ, 0.17)	<LOQ (<LOQ, <LOQ)
PFHxS	%>LOQ	100	100	100
	Median (Q1, Q3)	69.7 (58.0, 78.9)	11.6 (4.69, 22.8)	0.34 (0.30, 0.65)
PFHpS	%>LOQ	100	94	16
	Median (Q1, Q3)	4.18 (3.31, 4.94)	0.73 (0.29, 1.41)	<LOQ (<LOQ, <LOQ)
*tot-*PFOS	%>LOQ	100	100	100
	Median (Q1, Q3)	79.4 (62.3, 112.5)	18.2 (8.61, 31.8)	2.34 (1.92, 2.95)
*n-*PFOS	%>LOQ	100	100	100
	Median (Q1, Q3)	43.1 (31.2, 65.9)	11.5 (5.25, 19.7)	1.98 (1.52, 2.52)
*1m-*PFOS	%>LOQ	100	100	63
	Median (Q1, Q3)	8.89 (6.53, 10.9)	1.35 (0.63, 2.96)	0.11 (<LOQ, 0.14)
*2/6m-*PFOS	%>LOQ	100	100	100
	Median (Q1, Q3)	10.3 (7.79, 14.6)	2.14 (0.97, 3.96)	0.34 (0.29, 0.48)
*3/4/5m-*PFOS	%>LOQ	100	100	100
	Median (Q1, Q3)	20.5 (15.0, 25.4)	4.34 (1.75, 7.91)	0.40 (0.35, 0.56)
PFOA	%>LOQ	100	100	100
	Median (Q1, Q3)	5.65 (4.81, 7.50)	1.72 (1.09, 2.75)	0.98 (0.64, 1.37)
PFNA	%>LOQ	100	99	100
	Median (Q1, Q3)	0.51 (0.41, 0.66)	0.39 (0.28, 0.53)	0.41 (0.31, 0.50)
PFDA	%>LOQ	100	96	95
	Median (Q1, Q3)	0.25 (0.21, 0.34)	0.25 (0.19, 0.32)	0.23 (0.19, 0.31)
PFUnDA	%>LOQ	95	87	97
	Median (Q1, Q3)	0.22 (0.17, 0.33)	0.21 (0.14, 0.30)	0.20 (0.14, 0.31)

^1^LOQ = 0.1 ng/mL for all PFAS.

### Transplacental transfer efficiencies

**Fig. 2:**
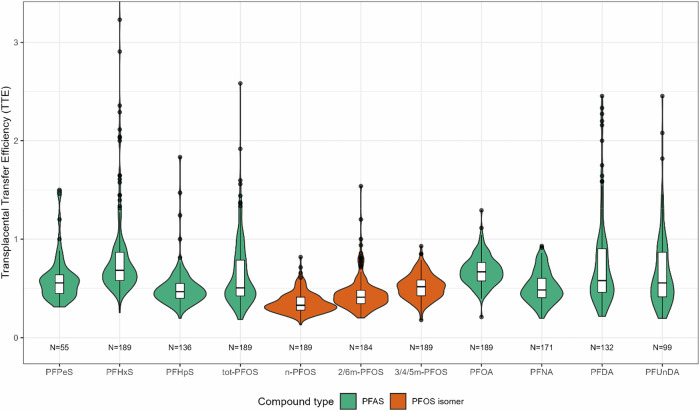
Violin plots of transplacental transfer efficiencies for PFAS compounds and PFOS isomers, estimated as the ratio of concentrations in the matched cord to maternal serum samples collected in pregnancy. An outlier TTE value for PFHxS (10.9) is outside the limit of the figure.

The highest TTE was observed for PFHxS (median TTE_mp:c_: 0.68) and PFOA (median TTE_mp:c_: 0.67) (Table 4 and Fig. 2). No clear patterns based on carbon chain length or active group were observed. However, branched PFOS isomers had higher TTE than linear PFOS. Substantial interindividual variation was observed, particularly for PFHxS but also for total PFOS, PFDA, and PFUnDA.

**Table 4:** Transplacental transfer efficiencies of PFAS estimated as the ratio of concentrations in matched cord to maternal serum samples collected in pregnancy (TTE_mp:c_), and at delivery (TTE_md:c_).

		TTE_mp:c_			TTE_md:c_	
PFAS compound/ PFOS isomer	*n*	Median (Q1, Q3)	Range	*n*	Median (Q1, Q3)	Range
PFPeS	55	0.56 (0.45, 0.64)	0.31–1.50	59	0.75 (0.61, 0.90)	0.43–1.36
PFHxS	189	0.68 (0.58, 0.87)	0.25–11.0	199	0.80 (0.68, 0.99)	0.28–8.67
PFHpS	136	0.47 (0.40, 0.55)	0.20–1.83	143	0.57 (0.48, 0.69)	0.23–1.94
*tot*-PFOS	189	0.51 (0.42, 0.79)	0.18–2.58	199	0.60 (0.49, 0.87)	0.21–3.72
*n*-PFOS	189	0.33 (0.28, 0.41)	0.13–0.82	199	0.38 (0.33, 0.46)	0.13–1.24
*2/6m*-PFOS	184	0.41 (0.34, 0.48)	0.20–1.54	194	0.47 (0.39, 0.57)	0.18–1.57
*3/4/5m*-PFOS	189	0.52 (0.43, 0.59)	0.18–0.93	199	0.57 (0.49, 0.67)	0.17–2.06
PFOA	189	0.67 (0.58, 0.76)	0.21–1.29	199	0.76 (0.67, 0.91)	0.22–2.09
PFNA	171	0.48 (0.41, 0.60)	0.20–0.93	179	0.56 (0.47, 0.66)	0.22–1.74
PFDA	132	0.58 (0.46, 0.90)	0.22–2.45	139	0.67 (0.50, 1.00)	0.14–2.73
PFUnDA	99	0.56 (0.41, 0.87)	0.20–2.45	102	0.60 (0.45, 0.79)	0.20–3.16

The calculations were restricted to samples with concentrations >LOQ (0.1 ng/mL for all PFAS).

TTEs were consistently higher for all compounds when estimated from maternal serum concentrations at delivery compared with maternal serum concentrations in pregnancy (Table 4).

When we stratified TTE_mp:c_ by exposure category, the estimates were higher in the background exposure category compared with the intermediate and high categories for PFHxS, total PFOS, and PFOS isomers (Table 5). In our regression models of TTE_mp:c_, TTE was significantly lower in the intermediate and high exposure categories compared to background exposure for all PFAS except for PFOA, even after accounting for other maternal characteristics (Table S3). For example, the estimated TTE_mp:c_ for PFHxS was 54.2 (95% CI: 61.3–45.9) percent lower in the high exposure category compared to the background exposure category.

**Table 5:** Transplacental transfer efficiencies estimated as the ratio of concentrations in matched cord to maternal serum samples collected in pregnancy, stratified by exposure category.

		Exposure category		
PFAS/PFOS isomer		High	Intermediate	Background
PFHxS	*n*	38	113	38
	Median (Q1, Q3)	0.60 (0.52, 0.69)	0.67 (0.58, 0.80)	1.27 (0.94, 1.65)
	Range	0.25–0.95	0.44–1.19	0.50–11.0
*tot-*PFOS	*n*	38	113	38
	Median (Q1, Q3)	0.42 (0.36, 0.49)	0.49 (0.42, 0.69)	1.00 (0.83, 1.26)
	Range	0.21–0.78	0.18–1.56	0.45–2.58
*n-*PFOS	*n*	38	113	38
	Median (Q1, Q3)	0.31 (0.27, 0.34)	0.33 (0.27, 0.39)	0.41 (0.35, 0.48)
	Range	0.13–0.45	0.20–0.82	0.24–0.71
*2/6m*-PFOS	*n*	38	112	34
	Median (Q1, Q3)	0.41 (0.33, 0.46)	0.40 (0.34, 0.47)	0.52 (0.40, 0.77)
	Range	0.21–0.53	0.23–0.80	0.20–1.54
*3/4/5m*-PFOS	*n*	38	113	38
	Median (Q1, Q3)	0.50 (0.42, 0.55)	0.49 (0.42, 0.58)	0.57 (0.50, 0.64)
	Range	0.18–0.85	0.29–0.85	0.33–0.93
PFOA	*n*	38	113	38
	Median (Q1, Q3)	0.66 (0.58, 0.74)	0.68 (0.59, 0.75)	0.62 (0.56, 0.80)
	Range	0.21–0.89	0.41–1.29	0.49–0.98

## Discussion

We assessed the TTE of nine PFAS and three PFOS isomers as the ratio between concentrations in umbilical cord serum and maternal serum in a birth cohort with a wide range of exposures to AFFF from contaminated drinking water in Ronneby, Sweden. Most previous research has studied populations exposed to substantially lower PFAS concentrations. The exposure scenario in Ronneby offers a unique setting to investigate transfer properties at higher levels of exposure, primarily to PFHxS and PFOS.

PFHxS and PFOA had the highest TTE but the transplacental transfer was overall of considerable magnitude with median TTEs between 0.40 and 0.68 for most PFAS. For PFOA, we did not observe any difference in TTE between the exposure categories, whereas both PFHxS and PFOS (total and isomer specific) showed higher TTE in the background exposed category, also after accounting for maternal characteristics. However, the wide range in TTE observed in the background exposure category for these compounds might be partly explained by possible interferences in cord serum samples [33], which have a higher relative impact at lower concentrations, and by the limited number of samples above the LOQ.

There is high variability in previously reported TTE of PFAS [13, 34]. This variation may partially be due to the timing of maternal blood sampling and small study populations. In addition, the estimated TTE may be impacted by interindividual variability and potentially also maternal exposure levels. Although these factors hamper comparisons between studies, some general patterns can be distinguished. Perfluorocarboxylic acids (PFCA) seem to cross the placental barrier more efficiently than PFSA when comparing compounds with the same carbon chain length [11, 25, 35, 36]. For example, the estimated TTE of PFOA is commonly reported as 0.8–1.3, whereas the TTE for PFOS is within the range of 0.3–0.46 [19, 31, 34, 37]. There seems to be a curvilinear trend with increasing chain length with a peak TTE for PFOA (C8) for PFCA and PFHxS (C6) for PFSA [11, 24]. Our findings agree with these results. In our study, PFHxS had the highest overall TTE among PFSA and PFOA had the highest overall TTE among PFCA. Furthermore, PFOA had the highest median TTE of all compounds in the intermediate and highly exposed categories.

Overall, our results confirm that PFAS compounds and PFOS isomers that are reported to have a lower binding affinity to serum albumin are more efficiently transported across the placenta i.e., PFOA vs. PFOS and branched PFOS vs. linear PFOS [20, 31, 38]. This suggests that it is mainly the unbound PFAS fraction that is transferred across the placenta through passive transport.

We observed a wide range of TTE for total PFOS and PFHxS in the background exposed category. The high variation may be explained by matrix interferences in cord serum (i.e., non-target analytes in a biological matrix), resulting in overestimated concentrations [33, 39, 40]. We observed analytical issues with some cord serum samples, especially in the analyses of *1m-*PFOS, PFHxS, and *tot-*PFOS, and we therefore decided not to include *1m-*PFOS in the TTE estimation. Interferences from endogenous compounds are likely included in the reported results of total PFOS in samples with low concentrations, although this concern is not specific to our study but applies to the whole research field. Issues with interferences and overestimation of concentrations of PFSA, in particular PFHxS and PFOS, are a general issue that has been highlighted by Chan et al. [39] and Benskin et al. [41] but the interferences that we observed in cord serum are not fully understood [33]. The concern primarily relates to serum samples in the low-exposure range, where it may cause systematic measurement errors. The relative error is smaller and of less concern for the interpretation of results at higher exposure levels. This is reflected in our study, as the TTEs for PFHxS and total PFOS and its isomers showed less variability in the intermediate- and high- exposure categories.

Previous studies are inconsistent regarding the sampling time of pregnancy serum. When we investigated the impact of the gestational stage at sample collection, we observed higher TTE for all compounds when the sample was collected at delivery (i.e., late) compared with samples collected during pregnancy. This result makes biological sense given the well-documented decrease in maternal serum PFAS concentrations throughout pregnancy due to an increase in maternal plasma volume and ongoing placental transfer [42]. It would be beneficial to use a standardized sampling protocol for both maternal and umbilical cord serum for assessing the TTE. At the minimum, future studies should always report the time of sampling, and variation in gestational stage at sampling should be considered when comparing results from different studies.

### Strengths and limitations

This is the first study of PFAS TTE in a highly exposed population. The exposure contrasts in the Ronneby Mother-Child Cohort allowed us to assess TTE at different exposure levels, which is a unique feature of this study. We provided all the sampling material used for sample collections, where all sample tubes were from the same batch, and all samples were analyzed in pairs to limit the risk of systematic bias in the chemical analysis.

One limitation of was the variation in gestational stage at pregnancy sampling. Most mothers in the background-exposed category were sampled in the third trimester when maternal serum PFAS concentrations are generally lower, which likely explains the higher TTE estimates in this category. When TTE is applied to understand the extent of prenatal exposure, it is preferable to use serum samples collected as early as possible in pregnancy to reflect maternal exposure levels at conception. However, this is often not feasible in practice. Thus, the most standardized sampling time is at delivery, and TTE estimates based on maternal delivery serum may be best suited for comparison between studies.

## Conclusions

The TTE of PFAS in matched maternal-cord serum samples from the Ronneby Mother-Child Cohort, covering a wide range of PFAS exposure dominated by PFHxS and PFOS, showed considerable transplacental transfer of most PFAS. The highest TTEs were observed for PFOA and PFHxS, and for branched PFOS isomers compared with linear. Although we observed lower TTEs in the high- and intermediate-exposure categories compared with the background-exposure category, the considerable TTE in highly exposed mothers implies high absolute fetal exposure. Further research is warranted to clarify the consequences of such high exposure during pregnancy for children in PFAS hotspots.

## Supplementary information

Supplementary checklist

Supplementary information

## Data Availability

The data contains sensitive personal data and cannot be shared.
