# Supplementary material for: Transplacental transfer efficiency of perfluoroalkyl substances (PFAS) after long-term exposure to highly contaminated drinking water: a study in the Ronneby Mother-Child Cohort
Source: J Expo Sci Environ Epidemiol. 2025 Mar 6;35(3):445–53. doi: 10.1038/s41370-025-00758-2 (PMC12069102; doi:10.1038/s41370-025-00758-2)

**Supplementary Information**

Transplacental transfer efficiency of perfluoroalkyl substances (PFAS) after long-term exposure to highly contaminated drinking water: A study in the Ronneby Mother-Child Cohort

***Erika Norén, Annelise J. Blomberg, Christian Lindh, Daniela Pineda, Kristina Jakobsson, and Christel Nielsen***

Table of Contents

[Analytical methods 2](#_Toc185335106)

[Supplementary Tables 4](#_Toc185335107)

[Table S1. Between-run and between-batch precision. The results of four quality control (QC) samples were used to calculate the between-run precision of the method, determined as the coefficient of variation (CV). The between-batch precision was determined by comparing duplicate analyses above the LOQ of 25% of the samples. 4](#_Toc185335108)

[Table S2. PFAS serum concentrations (ng/mL) in maternal serum collected at delivery and in cord serum, in the high, intermediate, and background exposure categories. 5](#_Toc185335109)

[Table S3. Results from the generalized mixed effects regression model of the change in TTE_mp:c_ in the high and intermediate exposure categories relative to the background-exposed category. Effect estimates are presented as the expected percent change in TTE relative to the background exposure group. 6](#_Toc185335110)

[Supplementary Figures 7](#_Toc185335111)

[Figure S1. Density plot illustrating the cut-offs for the three exposure categories based on measured PFHxS concentrations in maternal pregnancy serum. The cut-off for the background-exposure category was defined as the 90th percentile in the subgroup from Karlshamn (i.e., 0.90 ng/mL). The cut-off for the high-exposure category was set at the 75th percentile of all remaining women (i.e., 47.0 ng/mL). 7](#_Toc185335112)

[Figure S2. Spearman’s rank correlation coefficients for measured serum concentrations of PFAS compounds and PFOS isomers in maternal pregnancy and cord serum. 9](#_Toc185335113)

[Certificate of participation in G-EQUAS 10](#_Toc185335114)

# Analytical methods

Chemicals: All native (MPFAC-MXA, MP4HpA, and M5PFPeA) and isotopically labeled standards (MPFAC-MXA, M4 PFHpA, and M5 PFPeA) were purchased from Wellington Laboratories (Guelph, Ontario, Canada) as diluted reference standards. Acetonitrile, ammonium acetate, and methanol were from Merck (Darmstadt, Germany). Water was from a Milli-Q Integral 5 system (Millipore, Billerica, MA, USA).

Calibration standards, chemical blanks, and quality controls: Standard solutions were prepared by further dilution of diluted reference standards in water:acetonitrile (50:50). For the calibration standards, a blank matrix, Fetal Bovine Serum (FBS, Gibco, Thermo Fisher Scientific, Waltham, MA, USA) was used and prepared in the same way as the samples, except for the addition of 25 µl diluted standard solutions. Four reference samples were prepared for quality control (QC1, QC2, QC3, and QC4) by pooling serum samples containing different concentrations of the biomarkers (Table S3). Four QC samples and four chemical blanks (water), and calibration standards were included in the analysis of each sample batch (96-well plate).

Sample preparation of serum samples: The samples were prepared in 96-well plates with 2 ml flat-bottom glass vials (Biotech Solutions, Vineland, NJ, USA). Aliquots of 100 µl serum sample were added isotopically labeled internal standards. To precipitate the proteins, 200 µl of acetonitrile was added to all samples followed by vigorous shaking for 30 min. The samples were thereafter centrifuged at 2600g for 10 min. The supernatant (0.2 mL) was transferred to a new 96-well plate with 0.5 mL conical glass vials (MicroLiter Analytical Supplies, Inc., Suwanee, GA, USA) for analysis and again centrifuged at 3000g for 10min before analysis.

Analysis: For quantitative analysis a triple quadrupole linear ion trap mass spectrometers equipped with TurboIonSpray sources (QTRAP 5500, AB Sciex, Framingham, MA, USA) coupled to a liquid chromatography system (UFLCXR, Shimadzu Corporation, Kyoto, Japan; LC/MS/MS) was used. The MS analyses were carried out using selected reaction monitoring in negative ion mode. An aliquot of 4µl of the supernatant was injected on a C18 column (2.1 mm i.d. x 50 mm, Genesis Lightn; Hichrom, Reading, UK) used as a delay column for contaminating PFAS. The analytical column was an Acquity UPLC BEH C18 1.7 µm, 100*2.1mm (Waters, Wilmslow, UK). The mobile phases were A) 5mM ammonium acetate in water and B) methanol. The mobile phase was kept at 10% B for 1 min after injection. A gradient was then applied up to 95% B for 8 min, where it was kept for 1 min. The column was then conditioned at 10% B for 2 min. A diverter valve was used, and the column effluent was diverted to the MS between 5.0 and 10 min. The flow rate was 0.3 mL/min.

Quantification: The total, non-isomer specific compounds PFBS, PFPeS, PFHxS, PFHpS, PFOS, PFDS, PFPeA, PFHxA, PFHpA, PFOA, PFNA, PFDA, PFDoDA, PFUnDA, and PFTrDA were included in the analysis. The concentrations were determined by peak area ratios between the analyte and IS. At least two transitions were included for each PFAS in the analytical method, except for PFHxA and PFHxS. For higher specificity in the analysis of PFHxS, the ratios between three transitions (399-80, 399-99, and 399-119) were evaluated. In an additional analytical method described by Xu et al. (2020), PFOS was separated into linear PFOS (n-PFOS) and three separate isomer peaks. For the branched PFOS isomers, 2m-PFOS and 6m-PFOS could not be separated and the sum of perfluoro-2/6-methylheptanesulfonate (2/6m-PFOS) was evaluated using a calibration curve of 6m-PFOS. Likewise, the branched isomers 3m-PFOS, 4m-PFOS, and 5m-PFOS could not be separated, and therefore the sum of perfluoro-3/4/5-methylheptanesulfonate (3/4/5m-PFOS) was evaluated using a calibration curve for 5m-PFOS. The isomer perfluoro-1-methylheptanesulfonate (1m-PFOS) was omitted from further analysis because of suspected interferences. All values were corrected for the chemical blank.

Quality control and limit of quantification (LOQ): The LOQ was defined as ten times the standard deviation of the concentrations in chemical blank samples and was 0.1ng/mL for all PFAS. We estimated between-run precision using four quality control (QC) samples and between-batch precision by comparing results from duplicate sample analysis for 25% of the samples above LOQ (Table S1)

The laboratory participated successfully in the HBM4EU QA/QC program for PFAS analysis and participates bi-annually in the German External Quality Assessment Scheme (G-EQUAS) coordinated by the University of Erlangen-Nuremberg, Germany, for PFOA, PFNA, PFDA, PFBS, PFHpS, PFHxS, and PFOS analysis.

PFBS, PFDS, PFPeA, PFHxA, PFDoDA, and PFTrDA were analyzed but not quantified as the levels were not discernible and they were therefore not further assessed or included in the study.

# Supplementary Tables

Table S1. Between-run and between-batch precision. The results of four quality control (QC) samples were used to calculate the between-run precision of the method, determined as the coefficient of variation (CV). The between-batch precision was determined by comparing duplicate analyses above the LOQ of 25% of the samples.

| PFAS | QC1 (µg/L) | Between-run CV (%) | QC2 (µg/L) | Between-run CV (%) | QC3 (µg/L) | Between-run CV (%) | QC4 (µg/L) | Between-run CV (%) | Conc  (µg/L) | Between-batch CV (%) |
| --- | --- | --- | --- | --- | --- | --- | --- | --- | --- | --- |
| PFHpA | - | - | - | - | 0.7 | 14 | 128 | 8 | 0.2 | 42 |
| PFOA | 1.7 | 14 | 2.2 | 10 | 70 | 12 | 134 | 14 | 3.5 | 14 |
| PFNA | 1.4 | 17 | 1.4 | 12 | 2.0 | 15 | 131 | 14 | 0.4 | 16 |
| PFDA | 0.4 | 24 | 0.4 | 18 | 0.7 | 15 | 119 | 12 | 0.2 | 30 |
| PFUnDA | 0.5 | 20 | 0.5 | 20 | 0.6 | 18 | 114 | 13 | 0.2 | 33 |
| PFHxS | 8.8 | 8 | 15 | 8 | 916 | 7 | 109 | 8 | 32 | 8 |
| PFPeS | 0.2 | 20 | 0.3 | 17 | 35 | 12 | - | - | 0.9 | 7 |
| PFHpS | 0.5 | 23 | 0.8 | 17 | 75 | 13 | 0.3 | 28 | 1.8 | 15 |
| *tot-*PFOS | 13 | 11 | 19 | 8 | 1230 | 8 | 129 | 8 | 45 | 11 |
| *n-*PFOS | 7.5 | 6 | 11 | 7 | 686 | 6 | 127 | 6 | 25 | 14 |
| *3/4/5m-*PFOS | 2.6 | 13 | 4.1 | 13 | 272 | 9 | 2.4 | 20 | 9.3 | 22 |
| *2/6m-*PFOS | 1.5 | 20 | 2.5 | 22 | 68 | 13 | 1.5 | 23 | 5.5 | 16 |

## Table S2. PFAS serum concentrations (ng/mL) in maternal serum collected at delivery and in cord serum, in the high, intermediate, and background exposure categories.

|  |  | Exposure category | | |
| --- | --- | --- | --- | --- |
| PFAS/PFOS isomer |  | High | Intermediate | Background |
| ***Maternal serum at delivery*** | | ***n = 38*** | ***n = 112*** | ***n = 38*** |
| PFPeS | %>LOQ^1^ | 66 | 30 | 0 |
|  | Median (Q1, Q3) | 0.16 (<LOQ, 0.60) | <LOQ (<LOQ, 0.12) | <LOQ (<LOQ, <LOQ) |
| PFHxS | %>LOQ | 100 | 100 | 100 |
|  | Median (Q1, Q3) | 59.6 (50.4, 73.1) | 9.82 (3.83, 17.9) | 0.40 (0.30, 0.54) |
| PFHpS | %>LOQ | 97 | 95 | 16 |
|  | Median (Q1, Q3) | 3.30 (2.74, 4.34) | 0.60 (0.26, 1.18) | <LOQ (<LOQ, <LOQ) |
| *tot-*PFOS | %>LOQ | 100 | 100 | 100 |
|  | Median (Q1, Q3) | 66.5 (51.0, 98.1) | 15.5 (7.32, 30.1) | 2.27 (1.93, 2.86) |
| *n-*PFOS | %>LOQ | 100 | 100 | 100 |
|  | Median (Q1, Q3) | 32.8 (25.1, 55.1) | 9.51 (4.73, 16.2) | 1.75 (1.39, 2.42) |
| *1m-*PFOS | %>LOQ | 100 | 100 | 42 |
|  | Median (Q1, Q3) | 7.50 (5.96, 9.14) | 1.29 (0.56, 2.62) | <LOQ (<LOQ, 0.12) |
| *2/6m-*PFOS | %>LOQ | 100 | 100 | 100 |
|  | Median (Q1, Q3) | 8.14 (5.57, 12.6) | 1.81 (0.84, 3.50) | 0.34 (0.25, 0.46) |
| *3/4/5m-*PFOS | %>LOQ | 100 | 100 | 100 |
|  | Median (Q1, Q3) | 18.2 (13.3, 20.2) | 3.50 (1.55, 6.49) | 0.38 (0.32, 0.53) |
| PFOA | %>LOQ | 100 | 100 | 100 |
|  | Median (Q1, Q3) | 5.17 (3.64, 6.40) | 1.42 (1.02, 2.36) | 0.86 (0.54, 1.09) |
| PFNA | %>LOQ | 100 | 99 | 100 |
|  | Median (Q1, Q3) | 0.44 (0.31, 0.58) | 0.36 (0.25, 0.47) | 0.33 (0.29, 0.48) |
| PFDA | %>LOQ | 97 | 92 | 95 |
|  | Median (Q1, Q3) | 0.23 (0.17, 0.35) | 0.22 (0.15, 0.29) | 0.21 (0.15, 0.31) |
| PFUnDA | %>LOQ | 92 | 88 | 89 |
|  | Median (Q1, Q3) | 0.22 (0.16, 0.29) | 0.20 (0.12, 0.27) | 0.22 (0.15, 0.31) |
| ***Cord serum*** |  | ***n=38*** | ***n=113*** | ***n=38*** |
| PFPeS | %>LOQ | 71 | 31 | 8 |
|  | Median (Q1, Q3) | 0.14 (<LOQ, 0.38) | <LOQ (<LOQ, 0.10) | <LOQ (<LOQ, <LOQ) |
| PFHxS | %>LOQ | 100 | 100 | 100 |
|  | Median (Q1, Q3) | 38.2 (32.2, 54.7) | 6.97 (2.76, 14.4) | 0.51 (0.41, 0.65) |
| PFHpS | %>LOQ | 100 | 88 | 5 |
|  | Median (Q1, Q3) | 1.80 (1.40, 2.42) | 0.35 (0.15, 0.63) | <LOQ (<LOQ, <LOQ) |
| *tot-*PFOS | %>LOQ | 100 | 100 | 100 |
|  | Median (Q1, Q3) | 32.3 (24.8, 51.0) | 8.45 (5.16, 14.5) | 2.33 (1.94, 2.93) |
| *n-*PFOS | %>LOQ | 100 | 100 | 100 |
|  | Median (Q1, Q3) | 12.3 (9.09, 20.1) | 3.81 (1.85, 6.11) | 0.81 (0.64, 1.00) |
| *1m-*PFOS | %>LOQ | 100 | 100 | 42 |
|  | Median (Q1, Q3) | 6.37 (4.91, 7.96) | 1.04 (0.44, 2.25) | <LOQ (<LOQ, 0.12) |
| *2/6m-*PFOS | %>LOQ | 100 | 99 | 89 |
|  | Median (Q1, Q3) | 3.65 (2.60, 5.80) | 0.93 (0.38, 1.55) | 0.17 (0.13, 0.25) |
| *3/4/5m-*PFOS | %>LOQ | 100 | 100 | 100 |
|  | Median (Q1, Q3) | 9.16 (6.95, 13.5) | 1.99 (0.75, 3.87) | 0.24 (0.17, 0.29) |
| PFOA | %>LOQ | 100 | 100 | 100 |
|  | Median (Q1, Q3) | 3.64 (2.86, 5.11) | 1.21 (0.74, 1.77) | 0.65 (0.48, 0.90) |
| PFNA | %>LOQ | 97 | 90 | 87 |
|  | Median (Q1, Q3) | 0.26 (0.14, 0.37) | 0.19 (0.13, 0.26) | 0.18 (0.13, 0.23) |
| PFDA | %>LOQ | 79 | 71 | 68 |
|  | Median (Q1, Q3) | 0.16 (0.11, 0.22) | 0.14 (<LOQ, 0.19) | 0.11 (<LOQ, 0.18) |
| PFUnDA | %>LOQ | 55 | 56 | 50 |
|  | Median (Q1, Q3) | 0.11 (<LOQ, 0.15) | 0.11 (<LOQ, 0.16) | 0.09 (<LOQ, 0.13) |
| ^1^LOQ = 0.1 ng/mL for all PFAS. | |  |  |  |
|  |  |  |  |  |

## Table S3. Results from the generalized mixed effects regression model of the change in TTE_mp:c_ in the high and intermediate exposure categories relative to the background-exposed category. Effect estimates are presented as the expected percent change in TTE relative to the background exposure category.

| PFAS/PFOS-isomer | Exposure category | Effect estimate (95% CI) | |
| --- | --- | --- | --- |
|  |  | Unadjusted | Adjusted^a^ |
| PFHxS (n = 182) | High | -54.0 (-60.9, -46.0) | -54.2 (-61.3, -45.9) |
|  | Intermediate | -47.4 (-53.8, -40.0) | -47.3 (-53.9, -39.7) |
| *tot-*PFOS (n = 182) | High | -56.9 (-63.6, -48.9) | -57.6 (-64.3, -49.6) |
|  | Intermediate | -45.9 (-52.9, -37.9) | -46.4 (-53.5, -38.3) |
| *n-*PFOS (n = 182) | High | -26.9 (-35.1, -17.7) | -28.1 (-36.1, -19.1) |
|  | Intermediate | -20.8 (-28.1, -12.7) | -21.6 (-28.7, -13.7) |
| *2/6m-*PFOS (n = 178) | High | -32.7 (-41.3, -22.9) | -33.2 (-41.7, -23.5) |
|  | Intermediate | -29.5 (-37.0, -21.1) | -30.7 (-38.1, -22.4) |
| *3/4/5m-*PFOS (n = 182) | High | -16.2 (-25.3, -5.9) | -19.2 (-28.0, -9.3) |
|  | Intermediate | -12.7 (-20.5, -4.0) | -14.6 (-22.2, -6.2) |
| PFOA (n = 182) | High | -4.7 (-14.0, 5.7) | -5.0 (-14.1, 5.2) |
|  | Intermediate | 1.3 (-6.9, 10.2) | 0.52 (-7.4, 9.1) |

^a^Models are adjusted for the following maternal characteristics: parity, smoking in early pregnancy, maternal education, maternal age at delivery, and pre-pregnancy BMI.

# Supplementary Figures


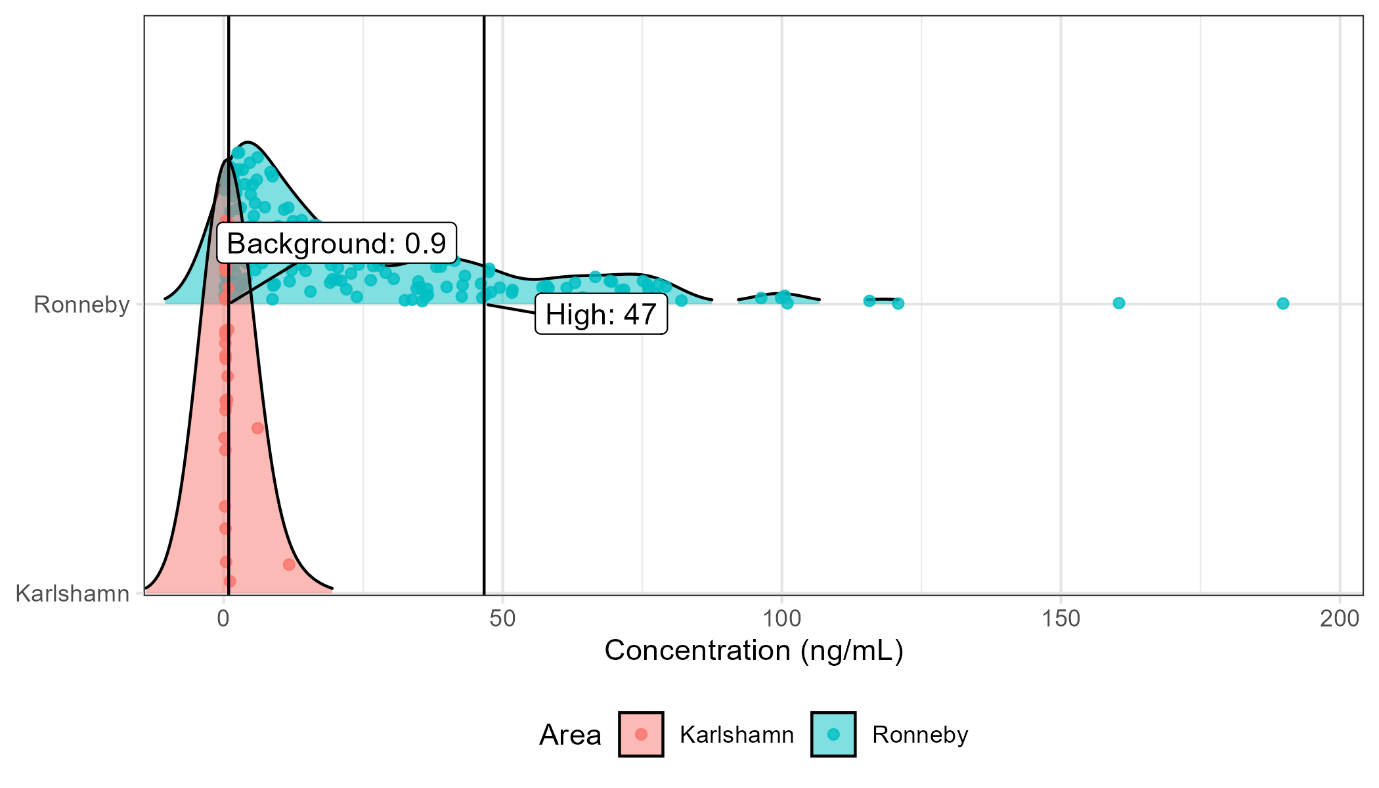


Figure S1. Density plot illustrating the cut-offs for the three exposure categories based on measured PFHxS concentrations in maternal pregnancy serum. The cut-off for the background exposure category was defined as the 90^th^ percentile in the subgroup from Karlshamn (i.e., 0.90 ng/mL). The cut-off for the high-exposure category was set at the 75^th^ percentile of all remaining women (i.e., 47 ng/mL).


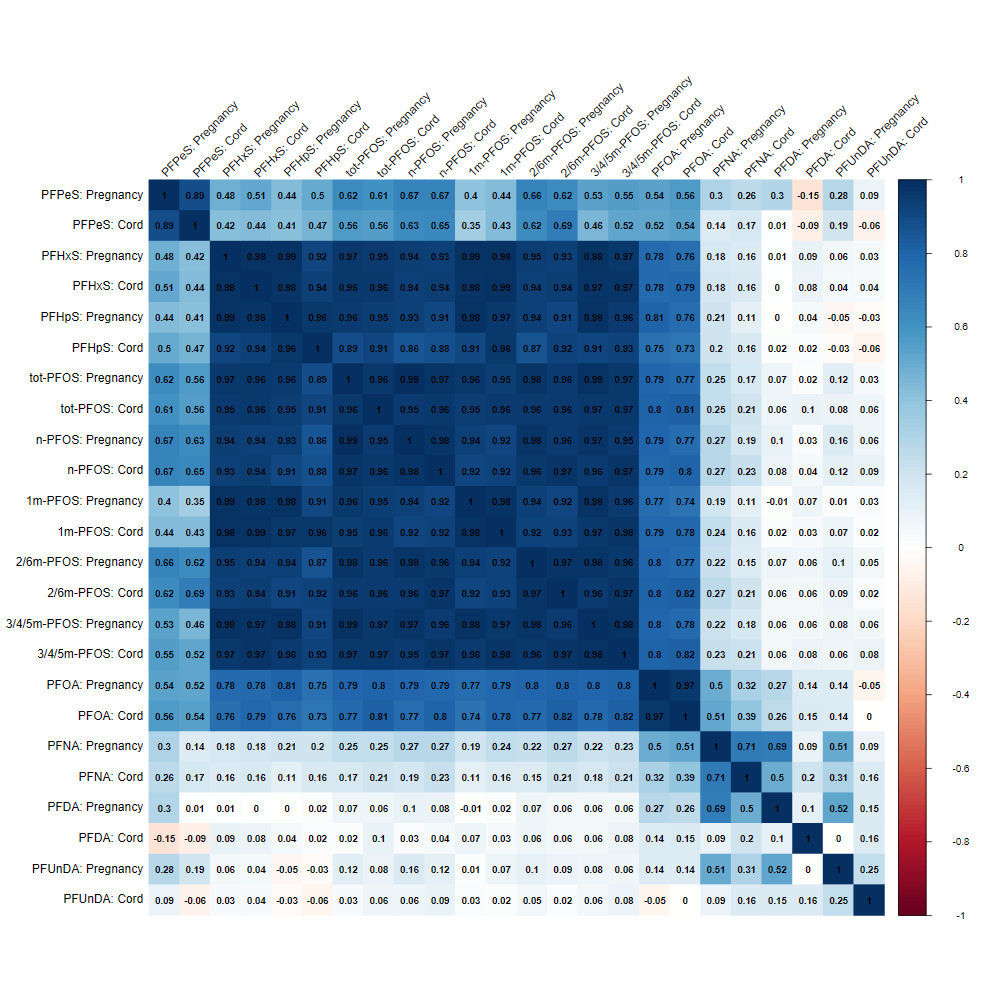


## Figure S2. Spearman’s rank correlation coefficients for measured serum concentrations of PFAS compounds and PFOS isomers in maternal pregnancy and cord serum.

# Certificate of participation in G-EQUAS


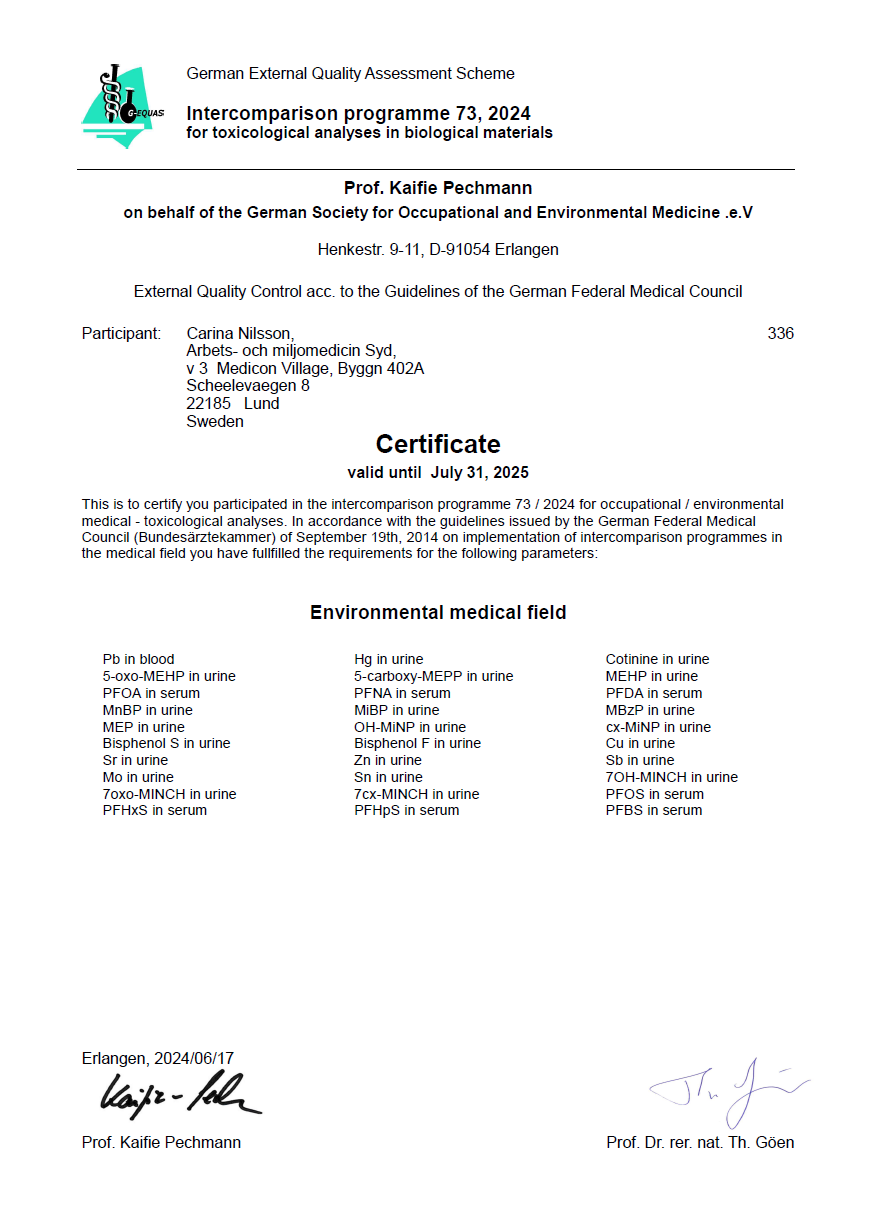

Supplement: Supplementary file 2 — Supplementary information [file 41370_2025_758_MOESM2_ESM.docx]
